# Impact of Grapefruit Juice on the Antiplatelet Activity of Loading and Maintenance Doses of Clopidogrel in Healthy Volunteers

**DOI:** 10.14740/cr307w

**Published:** 2014-02-27

**Authors:** Jennifer A. Campbell, Robyn Teply, Aryan N. Mooss, Daniel E. Hilleman

**Affiliations:** a Creighton University Cardiac Center, Omaha, Nebraska, USA

**Keywords:** Clopidogrel, Grapefruit juice, Platelets, Drug interactions

## Abstract

**Background:**

Grapefruit juice impacts the metabolism of a number of drugs via inhibition of a variety of metabolic enzymes. This study evaluated the impact of grapefruit juice on the antiplatelet activity of a loading dose and 7 days of maintenance therapy with clopidogrel.

**Methods:**

Healthy volunteers participated in two separate treatment protocols. The first protocol included a single 300 mg loading dose of clopidogrel and the second protocol included maintenance therapy with clopidogrel 75 mg given for 7 consecutive days. In both protocols, subjects were randomized to take clopidogrel with grapefruit juice or with tap water. At 6 h after the loading dose and at 6 h after the last maintenance dose of clopidogrel, a P2Y_12_ reaction unit (PRU) value using the VerifyNow^®^ P2Y12 assay was determined. A PRU value > 235 was defined as high on-treatment platelet reactivity (defined as clopidogrel hyporesponse).

**Results:**

Fourteen subjects completed the loading dose protocol while 17 subjects completed the maintenance dose protocol. Following administration of the loading dose, the mean PRUs with grapefruit juice and tap water were 235.2 (95% confidence interval (CI): 210.4-260.0) and 177.4 (95% CI: 141.6-213.2), respectively (P = 0.001). Following administration of the loading dose, the numbers of subjects with a PRU > 235 with grapefruit juice and tap water were 9 (64%) and 3 (21%), respectively (P = 0.05). In the maintenance dose protocol, the mean PRUs with grapefruit juice and tap water were 212.4 (95% CI: 175.8-249.0) and 186.1 (95% CI: 149.6-222.7), respectively (P = 0.059). In the maintenance dose protocol, the proportions of patients with a PRU > 235 with grapefruit juice and tap water were 9 (53%) and 4 (23%), respectively (P = 0.16).

**Discussion:**

Compared to tap water, grapefruit juice significantly increased the mean PRU in patients following a 300 mg loading dose of clopidogrel. The increase in mean PRU after 7 days of a 75 mg/day maintenance dose of clopidogrel was also increased by grapefruit juice, but the magnitude of the increase was not statistically significant. The proportion of subjects with high on-treatment platelet reactivity with clopidogrel after ingestion with grapefruit juice was not significant during either the loading or maintenance dose. The results of our study are insufficient to reach a valid conclusion concerning an interaction between clopidogrel and grapefruit juice.

## Introduction

Grapefruit juice may impact the metabolism of drugs via inhibition of cytochrome P-450 3A4 (CYP3A4), the cellular transport proteins P-glycoprotein (P-gp) and the organic anion-transporting polypeptides (OATPs), and intestinal esterases [1-4]. The magnitude of these interactions depends on a number of variables including the concentrations of furanocoumarins and flavanoids in the juice, the quantity of juice consumed, the duration of time over which the juice is consumed and the quantity of the affected enzymes in the patient [5].

Clopidogrel is a theinopyridine antiplatelet agent typically used alone or in combination with aspirin in patients with a variety of cardiovascular conditions [6]. Clopidogrel is a prodrug that undergoes a series of metabolic reactions to produce an active metabolite that blocks adenosine diphosphate binding to the P2Y_12_ platelet membrane receptor [7]. Approximately 85% of an orally administered clopidogrel dose is hydrolyzed by intestinal human carboxyl esterase-1 to an inactive carboxylic metabolite (SR26334) [7]. A portion of the remaining 15% is rapidly metabolized by cytochrome P450 isozymes in a two-step process to a highly labile active metabolite (R-130964) that forms a covalent irreversible bond with the P2Y_12_ receptor [7]. CYP2C19, CYP1A2 and CYP2B6 participate in the first metabolic step while CYP2C19, CYP2C9, CYP2B6 and CYP3A4 participate in the second metabolic step [8]. A number of drugs have been shown to reduce clopidogrel’s ability to inhibit platelet aggregation due to interactions involving CYP metabolism [9]. The Food and Drug Administration has recently advised that omeprazole, a CYP2C19 inhibitor, not be co-administered with clopidogrel [10].

Due to its ability to inhibit both intestinal esterase activity and CYP3A4, grapefruit juice has the potential to impact the antiplatelet activity of clopidogrel. While inhibition of CYP3A4 might decrease the antiplatelet activity of clopidogrel, inhibition of intestinal esterase may have the ability to increase the drug’s antiplatelet activity. The potential interaction between the ingestion of grapefruit juice and clopidogrel has not been investigated. The objective of the present study was to evaluate the interaction between grapefruit juice and the antiplatelet activity of clopidogrel in healthy volunteers during administration of a single 300 mg loading dose and following maintenance therapy with 75 mg/day for 7 days.

## Methods

### Subjects

This study was approved by the Creighton University Institutional Review Board. Healthy volunteers provided written informed consent and HIPAA waiver prior to participation. Healthy volunteers between the ages of 19 and 40 years were recruited to participate in the two treatment protocols (loading and maintenance doses). All volunteers were free of medical disease and were taking no medications. Screening procedures conducted within 3 days of study enrollment included a medical history and physical examination, complete blood count, serum chemical analysis (electrolytes, renal and hepatic function) and a urinalysis. Women underwent a pregnancy test prior to participation. Subjects were excluded if they had a history of medical or psychiatric disease, laboratory test abnormalities, a history of cigarette smoking, alcohol or illicit substance abuse, were greater than 25% above or below their ideal body weight, or had a history of habitual or recent ingestion of grapefruit juice.

### Study design

The study included two separate treatment protocols in which subjects receiving clopidogrel were randomized in cross-over fashion to receive the drug with grapefruit juice and with tap water. In one protocol, the effect of grapefruit juice on a single loading dose of clopidogrel 300 mg was studied. In the second protocol, the effect of grapefruit juice on a maintenance dose of clopidogrel 75 mg/day administered for 7 consecutive days was studied.

In the loading dose protocol, subjects were randomized to take a 300 mg dose of clopidogrel (4 × 75 mg Plavix^®^ tablets, Sanofi-Aventis US, Bridgewater, NJ) with either 240 mL of regular-strength white grapefruit juice or tap water. At 6 h after ingestion of clopidogrel, a 5 mL whole blood sample was drawn by venipuncture from the antecubital vein using a 21-gauge needle. Whole blood was collected in a blue-top plastic vacuette sodium citrate blood collection tube (Greiner, Monroe, NC). This blood was used to determine the P2Y_12_ reaction units (PRUs) using the VerifyNow^®^P2Y12 assay (Accumetrics, San Diego, California). After a minimum 2-week washout period, these subjects returned to the clinic to receive a second clopidogrel 300 mg dose receiving the alternate ingestion of grapefruit juice or tap water. A PRU value was again measured using methodology identical to that used during the initial clopidogrel administration.

In the maintenance dose protocol, subjects were randomized to receive clopidogrel 75 mg/day taking each daily dose with 240 mL of white grapefruit juice or with tap water for 7 consecutive days. A minimum 2-week washout period was required between the two treatment periods. On the seventh day of each treatment period at 6 h after the last clopidogrel dose, a PRU value was measured using the identical methodology as during the loading dose protocol.

### VerifyNow assay

The VerifyNow P2Y12 assay is a rapid, cartridge-based, point-of-care assay specifically measuring the direct effects of clopidogrel on the platelet P2Y_12_ receptor. The VerifyNow P2Y12 assay mimics turbidimetric aggregation. The cartridge contains fibrinogen-coated polystyrene beads and 20 µM adenosine diphosphate and 22 nM of prostaglandin E^1^. In a separate channel of the cartridge in which iso-TRAP is used as an agonist, a baseline value for platelet function is obtained. This enables assessment of inhibition of platelet aggregation without discontinuation of clopidogrel therapy. This assay reports the results as PRU. The tubes containing the whole blood were inverted 3-5 times and allowed to stand at room temperature for 10 min. The tubes were then inverted an additional 4-5 times and placed into the assay device. A PRU value > 235 was defined as high on-treatment platelet reactivity [11].

### Adverse effects

Although assessment of side effects was not a primary objective of this study, patients were asked to report any side effects at the time they occurred to one of the study investigators. Subjects were queried at each study visit about the occurrence of any side effect. Serious side effects were defined as those requiring discontinuation of clopidogrel, medical intervention, hospitalization, disability or death.

### Statistical analysis

Statistical analysis was performed using SAS (version 9.3; SAS Institute, Inc., Cary, NC). Continuous data were presented as the mean and standard deviation, and/or median where appropriate. Nominal data were presented as frequency count (*n*) and percentage (%). Since the distribution of differences in PRU values was not normal in the loading dose study, the non-parametric Wilcoxon signed rank test was used to compare the mean PRU values obtained during clopidogrel therapy taken with grapefruit juice and with tap water. As the distribution of the differences in PRU was normal in the maintenance dose study, a paired “t” test was used to compare the mean PRU values obtained during clopidogrel therapy taken with grapefruit juice and with tap water. The proportion of subjects with PRU values > 235 during clopidogrel therapy taken with grapefruit juice and with tap water for both the loading dose and maintenance dose studies was compared using the Fisher’s exact test. A P value < 0.05 was considered significant and all statistical tests were two-tailed.

## Results

### Subjects

A total of 35 (12 men and 23 women) healthy volunteers participated in this study. The original intent was to have 15 subjects complete the loading dose protocol and 20 subjects complete the maintenance dose protocol. Fourteen (five men and nine women) healthy volunteers aged 19 to 40 years (mean age 24.7 ± 6.2 years) completed the loading dose protocol. One volunteer in the loading dose study provided written informed consent but was lost to follow up prior to the first PRU value being obtained. Seventeen (four men and 13 women) healthy volunteers aged 19 to 38 years (mean age 25.5 ± 5.8 years) completed the maintenance dose protocol. Three additional volunteers gave consent to participate but did not complete the protocol and were lost to follow up. Two subjects completed the first assigned treatment and had a PRU value determined while the third subject failed to complete the first PRU analysis. Data from the four subjects who did not complete both PRU analyses were not included in the analysis.

### Antiplatelet activity

In the loading dose study, the mean ± 95% confidence intervals (CIs) for the PRU when clopidogrel was taken with grapefruit juice and tap water were 235.2 (95% CI: 210.4-235.2) and 177.4 (95% CI: 141.6-213.1), respectively (P = 0.001) (Table 1). Following the loading dose, high on-treatment platelet reactivity (PRU > 235) with grapefruit juice and tap water occurred in 64% (9/14) and 21% (3/14) of subjects, respectively (P = 0.05).

**Table 1:** PRU Values Following a Clopidogrel Loading Dose With Grapefruit Juice and With Tap Water

Patient	PRU	
	With grapefruit juice	With tap water
1	240+	227
2	172	101
3	290+	260+
4	161	140
5	250+	162
6	310+	260+
7	236+	89
8	253+	240+
9	225	207
10	232	100
11	260+	197
12	239+	234
13	255+	124
14	170	142
Mean (95% CI)*	235.2 (95% CI: 210.4 - 260.0)	177.4 (95% CI: 141.6 - 213.1)

*P = 0.001; + = clopidogrel hyporesponders; PRU: platelet resistance unit; CI: confidence interval.

In the maintenance dose protocol, the mean ± 95% CI PRUs with grapefruit juice and tap water were 212.4 (95% CI: 175.8-249.0) and 186.1 (95% CI: 149.6-222.7), respectively (P = 0.059) (Table 2). After 7 days of maintenance therapy, high on-treatment platelet reactivity with grapefruit juice and tap water occurred in 53% (9/17) and 23% (4/17) of subjects, respectively (P = 0.16).

**Table 2:** PRU Values Following Clopidogrel Maintenance Doses With Grapefruit Juice and With Tap Water

Patient	PRU	
	With grapefruit juice	With tap water
1	239+	92
2	299+	241+
3	74	80
4	245+	208
5	120	89
6	290+	237+
7	263+	340+
8	158	144
9	264+	170
10	99	130
11	254+	204
12	208	225
13	288+	296+
14	256+	169
15	227	206
16	126	144
17	201	189
Mean (95% CI)	212.4 (95% CI: 175.8 - 249.0)	186.1 (95% CI: 149.6 - 222.7)

*P = 0.059; + = clopidogrel hyporesponders; PRU: platelet resistance unit; CI: confidence interval.

### Adverse events

There was no significant difference in the number of subjects reporting side effects with clopidogrel therapy administered with tap water or with grapefruit juice during either treatment protocol (Table 3). No serious adverse events occurred during either treatment protocol. In the loading dose protocol, only one subject reported an adverse event. This subject complained of arthralgias following ingestion of clopidogrel 300 mg both with tap water and with grapefruit juice.

**Table 3:** Side Effects Reported During Maintenance Therapy in Subjects Receiving Clopidogrel With Grapefruit Juice and With Water

Subjects	Adverse events	
	Clopidogrel with water	Clopidogrel with grapefruit juice
1	Bruising	None
2	None	None
3	None	None
4	None	Bruising
5	None	None
6	Nausea	None
7	None	None
8	Diarrhea (1 episode)	None
9	None	Lower GI bleed (melena, dark tarry, stools × 2 days)
10	Diarrhea (1 episode)	None
11	Bruising; arthralgia	Arthralgia
12	None	None
13	None	Heartburn
14	Heartburn; headache	Bruising
15	Headache	Nausea
16	Epistaxis; headache	None
17	Heartburn	Heartburn
Total number of volunteers with adverse events	9 (53%)	7 (41%)

During the maintenance phase of the study, nine subjects taking clopidogrel with water and seven subjects taking clopidogrel with grapefruit juice reported side effects. Although no patient experienced a side effect during the maintenance protocol defined as serious, four subjects reported abnormal bruising, one subject reported epistaxis lasting 15 min and one subject reported the occurrence of black, tarry stools lasting 2 days that resolved without intervention.

## Discussion

Our study is the first to evaluate the effect of ingestion of grapefruit juice on the antiplatelet activity of clopidogrel. Grapefruit juice inhibits the antiplatelet activity of clopidogrel following both a single oral loading dose of 300 mg and after 7 days of the usual maintenance dose of 75 mg/day, but the difference in mean PRU values was significant only following the loading dose. This finding is of limited clinical relevance as it is highly unlikely that patients would be administered a loading dose of clopidogrel with grapefruit juice. There was a trend suggesting an effect of grapefruit juice on the mean PRU values with the maintenance dose of clopidogrel, but this difference did not reach statistical significance compared to ingestion with water.

Perhaps more importantly, the proportion of volunteers who had high on-treatment platelet reactivity (defined as a PRU > 235) was increased following ingestion of clopidogrel with grapefruit juice compared to tap water for both the loading dose and the maintenance dose. However, the differences in the proportions of volunteers with high on-treatment platelet reactivity with grapefruit juice failed to reach statistical significance. In clinical practice, it would be the PRU value that would be measured in individual patients to evaluate high on-treatment platelet reactivity during therapy with clopidogrel. With both the loading and maintenance clopidogrel doses, more than half of the volunteers would have been determined to have high on-treatment platelet reactivity when drug was ingested with grapefruit juice compared to about one in five patients when drug was ingested with water. Though not statistically significant, this finding could potentially lead to a greater number of patients taking clopidogrel with grapefruit juice being switched to a different antiplatelet agent.

Grapefruit juice has previously been demonstrated to interact pharmacokinetically with drugs metabolized by CYP3A4 [12]. Some of these interactions were of sufficient magnitude to produce adverse reactions. The most commonly reported interactions with grapefruit juice include statins and calcium channel blockers [12]. It has also been hypothesized that grapefruit juice may interact with the absorption and clearance of other drugs via inhibition of P-gp, OATPs and intestinal esterases [1-4]. These effects are less well established as a source of clinically relevant drug interactions.

Clopidogrel is a commonly used antiplatelet agent with a complex pharmacokinetic profile. It is a prodrug that is metabolized by intestinal esterases and CYP2C19 and CYP3A4 to an active metabolite that inhibits the platelet receptor P2Y_12_ [6-8]. It appears that CYP2C19 may be the most relevant rate-limiting step in the metabolism of clopidogrel as a number of studies suggest an interaction with CYP2C19 inhibitors may be clinically important. Certain proton pump inhibitors which inhibit CYP2C19 activity have been found to significantly inhibit the antiplatelet effects of clopidogrel [13-17].

Our study has a number of limitations. Our study may not have had adequate power to demonstrate a statistically significant effect of grapefruit juice on the antiplatelet effect of clopidogrel. It is also possible that grapefruit juice simply does not have a substantial or clinically important effect on the antiplatelet activity of clopidogrel. Our study also evaluated only 7 days of maintenance therapy with concomitant grapefruit juice and clopidogrel. Whether an interaction between clopidogrel and grapefruit juice would occur after a longer duration of therapy is unknown. Blood levels of clopidogrel and its active metabolites were also not measured. As a result, the nature of the potential interaction between clopidogrel and grapefruit juice could not be hypothesized. It has been well documented that patients with a genetic CYP2C19 loss of function allele have less inhibition of platelet aggregation with clopidogrel [18, 19]. Subjects in our study did not have an assessment of their CYP2C19 genetic metabolic status which may have affected our results. Finally, we only assessed the degree of inhibition of platelet aggregation using the VerifyNow P2Y12 assay. Many studies evaluating the antiplatelet effects of clopidogrel have used multiple methods to assess platelet activity. However, the VerifyNow P2Y12 assay has been assessed and verified to have accuracy similar to that of traditional optimal aggregometry [20, 21].

Given these limitations, the results of our study are insufficient to reach a valid conclusion concerning the effect of grapefruit juice on the antiplatelet activity of clopidogrel. Although the effect of grapefruit juice on the mean antiplatelet activity of the clopidogrel loading dose was significant, it is unlikely that grapefruit juice would be given with the loading dose of the drug. The increase in the proportion of patients with high on-treatment platelet reactivity when clopidogrel is taken with grapefruit juice is a cause for concern. Although it would seem prudent to avoid taking maintenance doses of clopidogrel with daily ingestion of grapefruit juice, the results of our study fall short of proving a definite evidence of an interaction. A larger number of subjects will need to be studied to determine if an interaction between grapefruit juice and clopidogrel actually exists.
